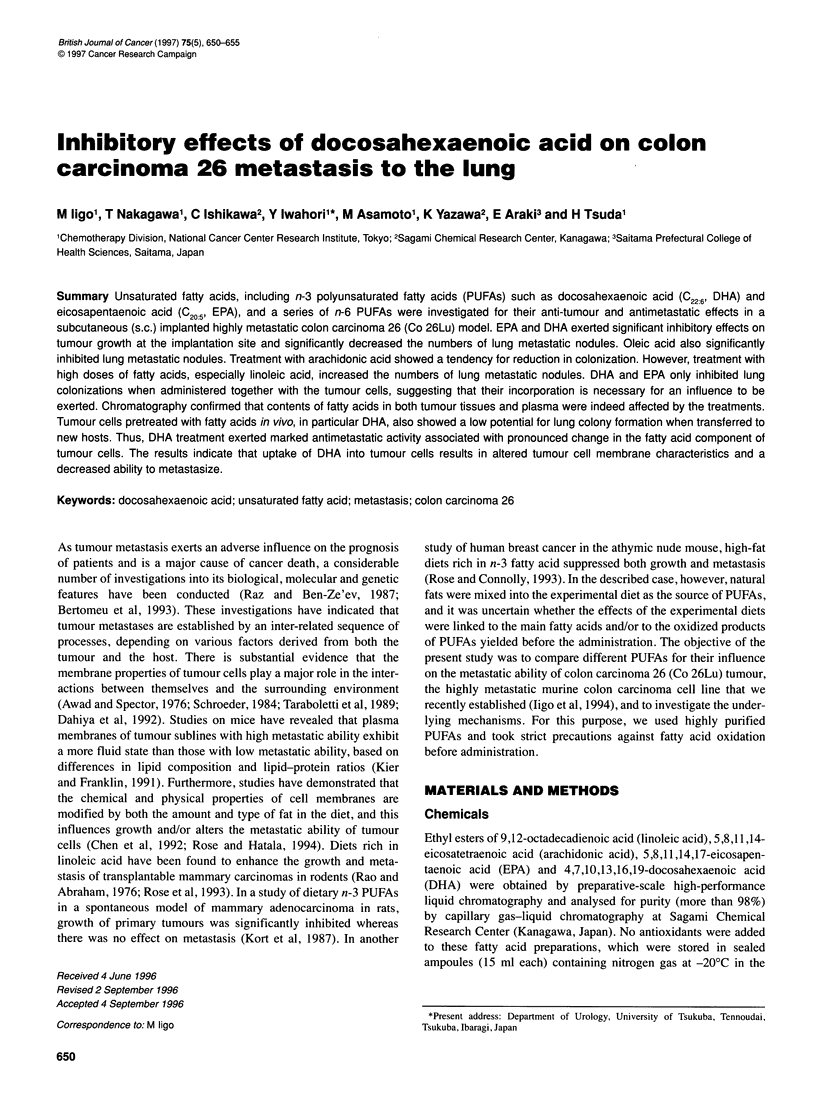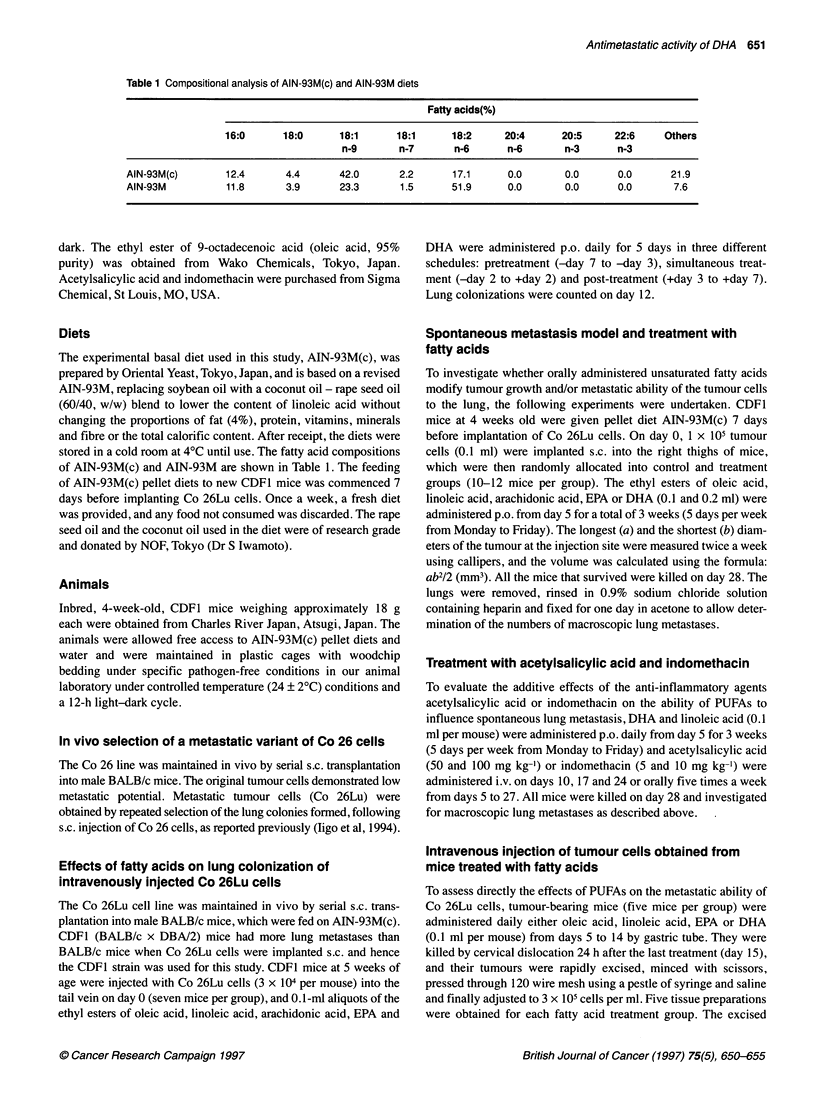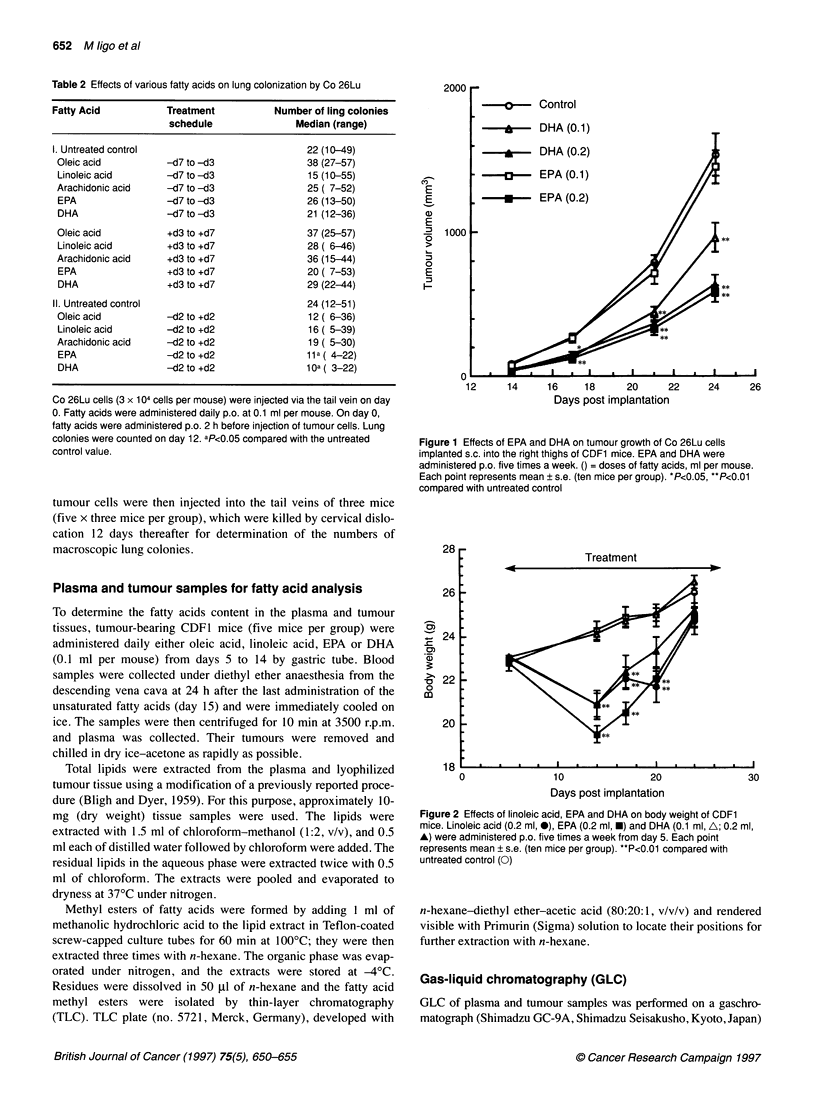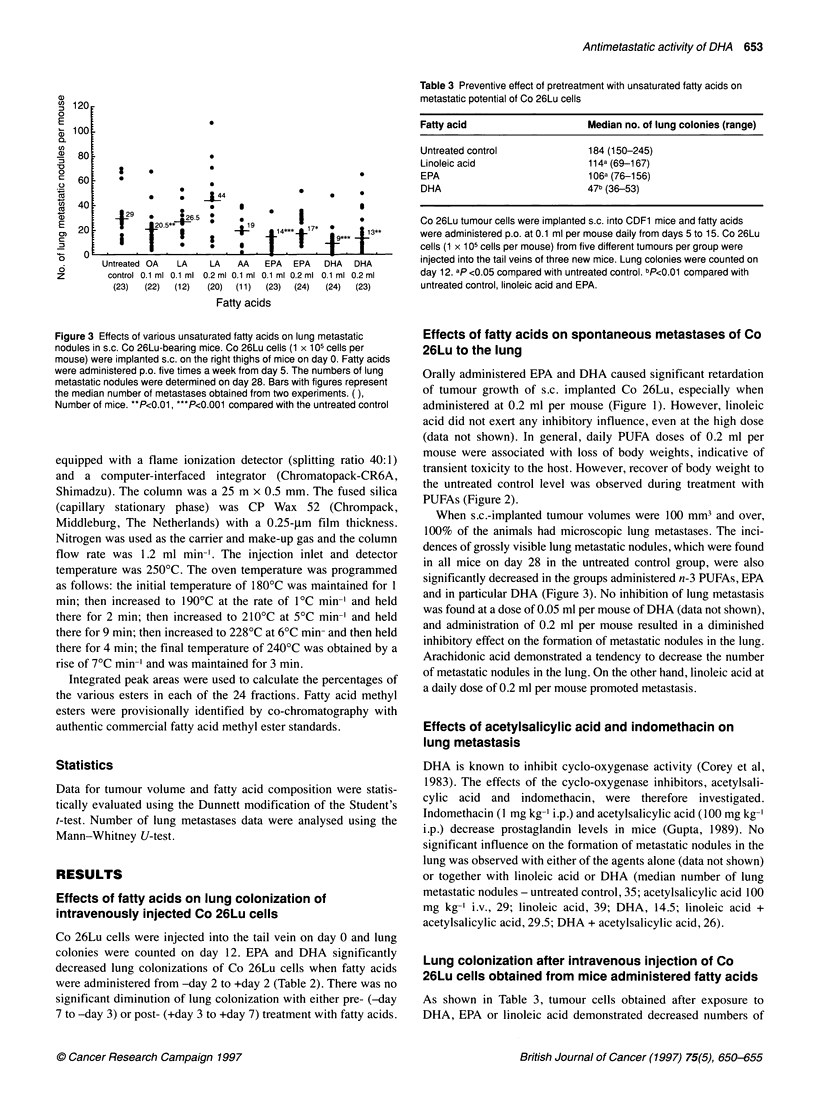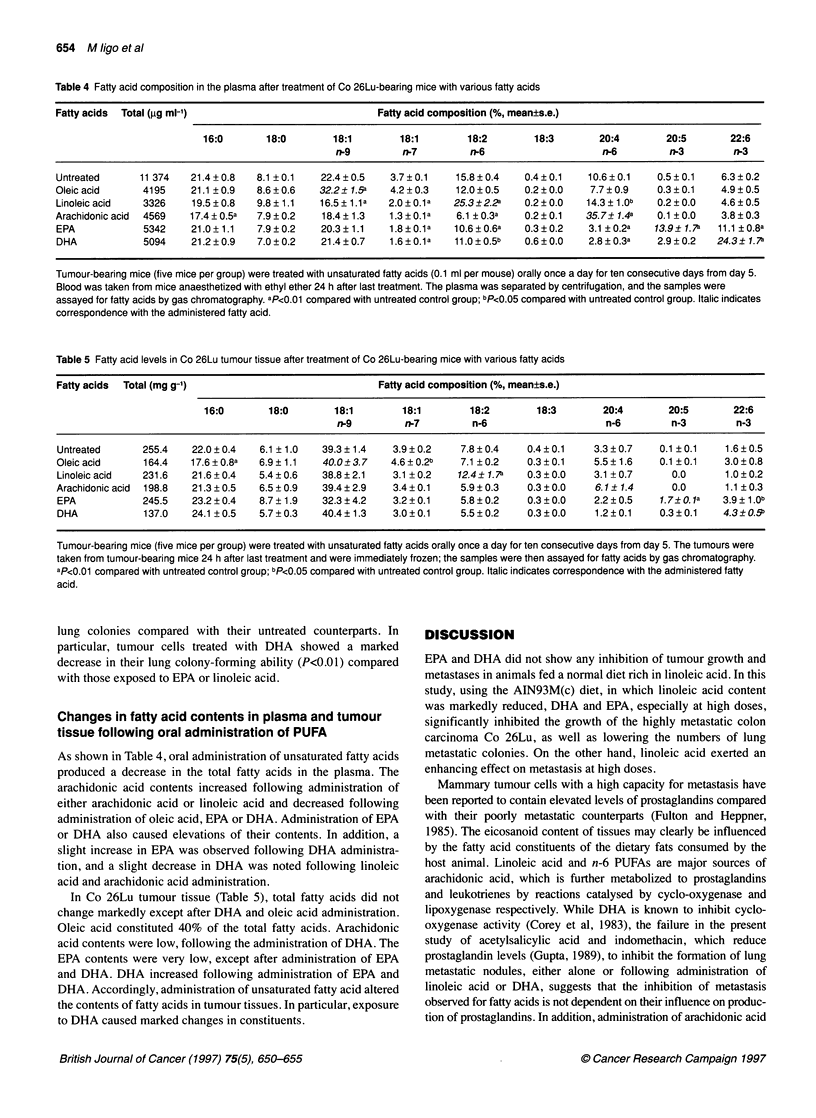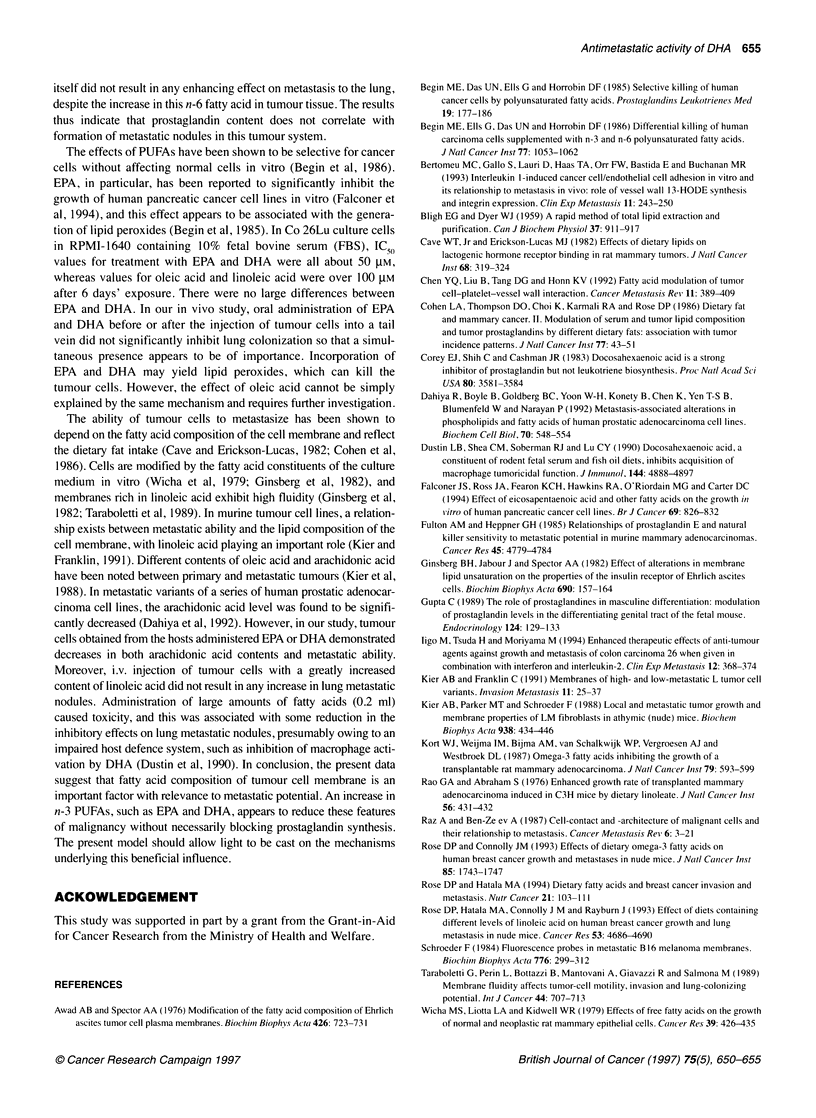# Inhibitory effects of docosahexaenoic acid on colon carcinoma 26 metastasis to the lung.

**DOI:** 10.1038/bjc.1997.116

**Published:** 1997

**Authors:** M. Iigo, T. Nakagawa, C. Ishikawa, Y. Iwahori, M. Asamoto, K. Yazawa, E. Araki, H. Tsuda

**Affiliations:** Chemotherapy Division, National Cancer Center Research Institute, Tokyo, Japan.

## Abstract

Unsaturated fatty acids, including n-3 polyunsaturated fatty acids (PUFAs) such as docosahexaenoic acid (C22:6, DHA) and eicosapentaenoic acid (C20:5, EPA), and a series of n-6 PUFAs were investigated for their anti-tumour and antimetastatic effects in a subcutaneous (s.c.) implanted highly metastatic colon carcinoma 26 (Co 26Lu) model. EPA and DHA exerted significant inhibitory effects on tumour growth at the implantation site and significantly decreased the numbers of lung metastatic nodules. Oleic acid also significantly inhibited lung metastatic nodules. Treatment with arachidonic acid showed a tendency for reduction in colonization. However, treatment with high doses of fatty acids, especially linoleic acid, increased the numbers of lung metastatic nodules. DHA and EPA only inhibited lung colonizations when administered together with the tumour cells, suggesting that their incorporation is necessary for an influence to be exerted. Chromatography confirmed that contents of fatty acids in both tumour tissues and plasma were indeed affected by the treatments. Tumour cells pretreated with fatty acids in vivo, in particular DHA, also showed a low potential for lung colony formation when transferred to new hosts. Thus, DHA treatment exerted marked antimetastatic activity associated with pronounced change in the fatty acid component of tumour cells. The results indicate that uptake of DHA into tumour cells results in altered tumour cell membrane characteristics and a decreased ability to metastasize.


British Journal of Cancer (1997) 75(5), 650–655
© 1997 Cancer Research Campaign

### Inhibitory effects of docosahexaenoic acid on colon carcinoma 26 metastasis to the lung

**M Iigo[1], T Nakagawa[1], C Ishikawa[2], Y Iwahori[1]*, M Asamoto[1], K Yazawa[2], E Araki[3] and H Tsuda[1]**

[1]Chemotherapy Division, National Cancer Center Research Institute, Tokyo; [2]Sagami Chemical Research Center, Kanagawa; [3]Saitama Prefectural College of Health Sciences, Saitama, Japan

**Summary** Unsaturated fatty acids, including *n*-3 polyunsaturated fatty acids (PUFAs) such as docosahexaenoic acid ($C_{22:6}$, DHA) and eicosapentaenoic acid ($C_{20:5}$, EPA), and a series of *n*-6 PUFAs were investigated for their anti-tumour and antimetastatic effects in a subcutaneous (s.c.) implanted highly metastatic colon carcinoma 26 (Co 26Lu) model. EPA and DHA exerted significant inhibitory effects on tumour growth at the implantation site and significantly decreased the numbers of lung metastatic nodules. Oleic acid also significantly inhibited lung metastatic nodules. Treatment with arachidonic acid showed a tendency for reduction in colonization. However, treatment with high doses of fatty acids, especially linoleic acid, increased the numbers of lung metastatic nodules. DHA and EPA only inhibited lung colonizations when administered together with the tumour cells, suggesting that their incorporation is necessary for an influence to be exerted. Chromatography confirmed that contents of fatty acids in both tumour tissues and plasma were indeed affected by the treatments. Tumour cells pretreated with fatty acids *in vivo*, in particular DHA, also showed a low potential for lung colony formation when transferred to new hosts. Thus, DHA treatment exerted marked antimetastatic activity associated with pronounced change in the fatty acid component of tumour cells. The results indicate that uptake of DHA into tumour cells results in altered tumour cell membrane characteristics and a decreased ability to metastasize.

**Keywords:** docosahexaenoic acid; unsaturated fatty acid; metastasis; colon carcinoma 26

As tumour metastasis exerts an adverse influence on the prognosis of patients and is a major cause of cancer death, a considerable number of investigations into its biological, molecular and genetic features have been conducted (Raz and Ben-Ze'ev, 1987; Bertomeu et al, 1993). These investigations have indicated that tumour metastases are established by an inter-related sequence of processes, depending on various factors derived from both the tumour and the host. There is substantial evidence that the membrane properties of tumour cells play a major role in the interactions between themselves and the surrounding environment (Awad and Spector, 1976; Schroeder, 1984; Taraboletti et al, 1989; Dahiya et al, 1992). Studies on mice have revealed that plasma membranes of tumour sublines with high metastatic ability exhibit a more fluid state than those with low metastatic ability, based on differences in lipid composition and lipid–protein ratios (Kier and Franklin, 1991). Furthermore, studies have demonstrated that the chemical and physical properties of cell membranes are modified by both the amount and type of fat in the diet, and this influences growth and/or alters the metastatic ability of tumour cells (Chen et al, 1992; Rose and Hatala, 1994). Diets rich in linoleic acid have been found to enhance the growth and metastasis of transplantable mammary carcinomas in rodents (Rao and Abraham, 1976; Rose et al, 1993). In a study of dietary *n*-3 PUFAs in a spontaneous model of mammary adenocarcinoma in rats, growth of primary tumours was significantly inhibited whereas there was no effect on metastasis (Kort et al, 1987). In another study of human breast cancer in the athymic nude mouse, high-fat diets rich in *n*-3 fatty acid suppressed both growth and metastasis (Rose and Connolly, 1993). In the described case, however, natural fats were mixed into the experimental diet as the source of PUFAs, and it was uncertain whether the effects of the experimental diets were linked to the main fatty acids and/or to the oxidized products of PUFAs yielded before the administration. The objective of the present study was to compare different PUFAs for their influence on the metastatic ability of colon carcinoma 26 (Co 26Lu) tumour, the highly metastatic murine colon carcinoma cell line that we recently established (Iigo et al, 1994), and to investigate the underlying mechanisms. For this purpose, we used highly purified PUFAs and took strict precautions against fatty acid oxidation before administration.

*Received 4 June 1996*
*Revised 2 September 1996*
*Accepted 4 September 1996*

*Correspondence to:* M Iigo

### MATERIALS AND METHODS

### Chemicals

Ethyl esters of 9,12-octadecadienoic acid (linoleic acid), 5,8,11,14-eicosatetraenoic acid (arachidonic acid), 5,8,11,14,17-eicosapentaenoic acid (EPA) and 4,7,10,13,16,19-docosahexaenoic acid (DHA) were obtained by preparative-scale high-performance liquid chromatography and analysed for purity (more than 98%) by capillary gas–liquid chromatography at Sagami Chemical Research Center (Kanagawa, Japan). No antioxidants were added to these fatty acid preparations, which were stored in sealed ampoules (15 ml each) containing nitrogen gas at –20°C in the

*Present address: Department of Urology, University of Tsukuba, Tennoudai, Tsukuba, Ibaragi, Japan

**Table 1** Compositional analysis of AIN-93M(c) and AIN-93M diets

| | Fatty acids(%) | | | | | | | | |
|---|---|---|---|---|---|---|---|---|---|
| | 16:0 | 18:0 | 18:1 n-9 | 18:1 n-7 | 18:2 n-6 | 20:4 n-6 | 20:5 n-3 | 22:6 n-3 | Others |
| AIN-93M(c) | 12.4 | 4.4 | 42.0 | 2.2 | 17.1 | 0.0 | 0.0 | 0.0 | 21.9 |
| AIN-93M | 11.8 | 3.9 | 23.3 | 1.5 | 51.9 | 0.0 | 0.0 | 0.0 | 7.6 |

dark. The ethyl ester of 9-octadecenoic acid (oleic acid, 95% purity) was obtained from Wako Chemicals, Tokyo, Japan. Acetylsalicylic acid and indomethacin were purchased from Sigma Chemical, St Louis, MO, USA.

### Diets

The experimental basal diet used in this study, AIN-93M(c), was prepared by Oriental Yeast, Tokyo, Japan, and is based on a revised AIN-93M, replacing soybean oil with a coconut oil – rape seed oil (60/40, w/w) blend to lower the content of linoleic acid without changing the proportions of fat (4%), protein, vitamins, minerals and fibre or the total calorific content. After receipt, the diets were stored in a cold room at 4°C until use. The fatty acid compositions of AIN-93M(c) and AIN-93M are shown in Table 1. The feeding of AIN-93M(c) pellet diets to new CDF1 mice was commenced 7 days before implanting Co 26Lu cells. Once a week, a fresh diet was provided, and any food not consumed was discarded. The rape seed oil and the coconut oil used in the diet were of research grade and donated by NOF, Tokyo (Dr S Iwamoto).

### Animals

Inbred, 4-week-old, CDF1 mice weighing approximately 18 g each were obtained from Charles River Japan, Atsugi, Japan. The animals were allowed free access to AIN-93M(c) pellet diets and water and were maintained in plastic cages with woodchip bedding under specific pathogen-free conditions in our animal laboratory under controlled temperature (24 ± 2°C) conditions and a 12-h light–dark cycle.

### In vivo selection of a metastatic variant of Co 26 cells

The Co 26 line was maintained in vivo by serial s.c. transplantation into male BALB/c mice. The original tumour cells demonstrated low metastatic potential. Metastatic tumour cells (Co 26Lu) were obtained by repeated selection of the lung colonies formed, following s.c. injection of Co 26 cells, as reported previously (Iigo et al, 1994).

### Effects of fatty acids on lung colonization of intravenously injected Co 26Lu cells

The Co 26Lu cell line was maintained in vivo by serial s.c. transplantation into male BALB/c mice, which were fed on AIN-93M(c). CDF1 (BALB/c × DBA/2) mice had more lung metastases than BALB/c mice when Co 26Lu cells were implanted s.c. and hence the CDF1 strain was used for this study. CDF1 mice at 5 weeks of age were injected with Co 26Lu cells ($3 \times 10^4$ per mouse) into the tail vein on day 0 (seven mice per group), and 0.1-ml aliquots of the ethyl esters of oleic acid, linoleic acid, arachidonic acid, EPA and DHA were administered p.o. daily for 5 days in three different schedules: pretreatment (–day 7 to –day 3), simultaneous treatment (–day 2 to +day 2) and post-treatment (+day 3 to +day 7). Lung colonizations were counted on day 12.

### Spontaneous metastasis model and treatment with fatty acids

To investigate whether orally administered unsaturated fatty acids modify tumour growth and/or metastatic ability of the tumour cells to the lung, the following experiments were undertaken. CDF1 mice at 4 weeks old were given pellet diet AIN-93M(c) 7 days before implantation of Co 26Lu cells. On day 0, $1 \times 10^5$ tumour cells (0.1 ml) were implanted s.c. into the right thighs of mice, which were then randomly allocated into control and treatment groups (10–12 mice per group). The ethyl esters of oleic acid, linoleic acid, arachidonic acid, EPA or DHA (0.1 and 0.2 ml) were administered p.o. from day 5 for a total of 3 weeks (5 days per week from Monday to Friday). The longest ($a$) and the shortest ($b$) diameters of the tumour at the injection site were measured twice a week using callipers, and the volume was calculated using the formula: $ab^2/2$ (mm$^3$). All the mice that survived were killed on day 28. The lungs were removed, rinsed in 0.9% sodium chloride solution containing heparin and fixed for one day in acetone to allow determination of the numbers of macroscopic lung metastases.

### Treatment with acetylsalicylic acid and indomethacin

To evaluate the additive effects of the anti-inflammatory agents acetylsalicylic acid or indomethacin on the ability of PUFAs to influence spontaneous lung metastasis, DHA and linoleic acid (0.1 ml per mouse) were administered p.o. daily from day 5 for 3 weeks (5 days per week from Monday to Friday) and acetylsalicylic acid (50 and 100 mg kg$^{-1}$) or indomethacin (5 and 10 mg kg$^{-1}$) were administered i.v. on days 10, 17 and 24 or orally five times a week from days 5 to 27. All mice were killed on day 28 and investigated for macroscopic lung metastases as described above.

### Intravenous injection of tumour cells obtained from mice treated with fatty acids

To assess directly the effects of PUFAs on the metastatic ability of Co 26Lu cells, tumour-bearing mice (five mice per group) were administered daily either oleic acid, linoleic acid, EPA or DHA (0.1 ml per mouse) from days 5 to 14 by gastric tube. They were killed by cervical dislocation 24 h after the last treatment (day 15), and their tumours were rapidly excised, minced with scissors, pressed through 120 wire mesh using a pestle of syringe and saline and finally adjusted to $3 \times 10^5$ cells per ml. Five tissue preparations were obtained for each fatty acid treatment group. The excised

© Cancer Research Campaign 1997

Table 2 Effects of various fatty acids on lung colonization by Co 26Lu

| Fatty Acid | Treatment schedule | Number of ling colonies Median (range) |
|---|---|---|
| I. Untreated control | | 22 (10–49) |
| Oleic acid | –d7 to –d3 | 38 (27–57) |
| Linoleic acid | –d7 to –d3 | 15 (10–55) |
| Arachidonic acid | –d7 to –d3 | 25 ( 7–52) |
| EPA | –d7 to –d3 | 26 (13–50) |
| DHA | –d7 to –d3 | 21 (12–36) |
| Oleic acid | +d3 to +d7 | 37 (25–57) |
| Linoleic acid | +d3 to +d7 | 28 ( 6–46) |
| Arachidonic acid | +d3 to +d7 | 36 (15–44) |
| EPA | +d3 to +d7 | 20 ( 7–53) |
| DHA | +d3 to +d7 | 29 (22–44) |
| II. Untreated control | | 24 (12–51) |
| Oleic acid | –d2 to +d2 | 12 ( 6–36) |
| Linoleic acid | –d2 to +d2 | 16 ( 5–39) |
| Arachidonic acid | –d2 to +d2 | 19 ( 5–30) |
| EPA | –d2 to +d2 | 11[a] ( 4–22) |
| DHA | –d2 to +d2 | 10[a] ( 3–22) |

Co 26Lu cells (3 x $10^4$ cells per mouse) were injected via the tail vein on day 0. Fatty acids were administered daily p.o. at 0.1 ml per mouse. On day 0, fatty acids were administered p.o. 2 h before injection of tumour cells. Lung colonies were counted on day 12. [a]*P*<0.05 compared with the untreated control value.

Figure 1 Effects of EPA and DHA on tumour growth of Co 26Lu cells implanted s.c. into the right thighs of CDF1 mice. EPA and DHA were administered p.o. five times a week. () = doses of fatty acids, ml per mouse. Each point represents mean ± s.e. (ten mice per group). **P*<0.05, ***P*<0.01 compared with untreated control tumour cells were then injected into the tail veins of three mice (five x three mice per group), which were killed by cervical dislocation 12 days thereafter for determination of the numbers of macroscopic lung colonies.

Figure 2 Effects of linoleic acid, EPA and DHA on body weight of CDF1 mice. Linoleic acid (0.2 ml, ●), EPA (0.2 ml, ■) and DHA (0.1 ml, △; 0.2 ml, ▲) were administered p.o. five times a week from day 5. Each point represents mean ± s.e. (ten mice per group). **P<0.01 compared with untreated control (○)

### Plasma and tumour samples for fatty acid analysis

To determine the fatty acids content in the plasma and tumour tissues, tumour-bearing CDF1 mice (five mice per group) were administered daily either oleic acid, linoleic acid, EPA or DHA (0.1 ml per mouse) from days 5 to 14 by gastric tube. Blood samples were collected under diethyl ether anaesthesia from the descending vena cava at 24 h after the last administration of the unsaturated fatty acids (day 15) and were immediately cooled on ice. The samples were then centrifuged for 10 min at 3500 r.p.m. and plasma was collected. Their tumours were removed and chilled in dry ice–acetone as rapidly as possible.

Total lipids were extracted from the plasma and lyophilized tumour tissue using a modification of a previously reported procedure (Bligh and Dyer, 1959). For this purpose, approximately 10-mg (dry weight) tissue samples were used. The lipids were extracted with 1.5 ml of chloroform–methanol (1:2, v/v), and 0.5 ml each of distilled water followed by chloroform were added. The residual lipids in the aqueous phase were extracted twice with 0.5 ml of chloroform. The extracts were pooled and evaporated to dryness at 37°C under nitrogen.

Methyl esters of fatty acids were formed by adding 1 ml of methanolic hydrochloric acid to the lipid extract in Teflon-coated screw-capped culture tubes for 60 min at 100°C; they were then extracted three times with *n*-hexane. The organic phase was evaporated under nitrogen, and the extracts were stored at –4°C. Residues were dissolved in 50 µl of *n*-hexane and the fatty acid methyl esters were isolated by thin-layer chromatography (TLC). TLC plate (no. 5721, Merck, Germany), developed with *n*-hexane–diethyl ether–acetic acid (80:20:1, v/v/v) and rendered visible with Primurin (Sigma) solution to locate their positions for further extraction with *n*-hexane.

### Gas-liquid chromatography (GLC)

GLC of plasma and tumour samples was performed on a gaschromatograph (Shimadzu GC-9A, Shimadzu Seisakusho, Kyoto, Japan)

© Cancer Research Campaign 1997

**Figure 3** Effects of various unsaturated fatty acids on lung metastatic nodules in s.c. Co 26Lu-bearing mice. Co 26Lu cells (1 × $10^5$ cells per mouse) were implanted s.c. on the right thighs of mice on day 0. Fatty acids were administered p.o. five times a week from day 5. The numbers of lung metastatic nodules were determined on day 28. Bars with figures represent the median number of metastases obtained from two experiments. ( ), Number of mice. $^{**}P$<0.01, $^{***}P$<0.001 compared with the untreated control equipped with a flame ionization detector (splitting ratio 40:1) and a computer-interfaced integrator (Chromatopack-CR6A, Shimadzu). The column was a 25 m × 0.5 mm. The fused silica (capillary stationary phase) was CP Wax 52 (Chrompack, Middleburg, The Netherlands) with a 0.25-μm film thickness. Nitrogen was used as the carrier and make-up gas and the column flow rate was 1.2 ml $min^{-1}$. The injection inlet and detector temperature was 250°C. The oven temperature was programmed as follows: the initial temperature of 180°C was maintained for 1 min; then increased to 190°C at the rate of 1°C $min^{-1}$ and held there for 2 min; then increased to 210°C at 5°C $min^{-1}$ and held there for 9 min; then increased to 228°C at 6°C $min^{-}$ and then held there for 4 min; the final temperature of 240°C was obtained by a rise of 7°C $min^{-1}$ and was maintained for 3 min.

Integrated peak areas were used to calculate the percentages of the various esters in each of the 24 fractions. Fatty acid methyl esters were provisionally identified by co-chromatography with authentic commercial fatty acid methyl ester standards.

### Statistics

Data for tumour volume and fatty acid composition were statistically evaluated using the Dunnett modification of the Student's *t*-test. Number of lung metastases data were analysed using the Mann–Whitney *U*-test.

### RESULTS

### Effects of fatty acids on lung colonization of intravenously injected Co 26Lu cells

Co 26Lu cells were injected into the tail vein on day 0 and lung colonies were counted on day 12. EPA and DHA significantly decreased lung colonizations of Co 26Lu cells when fatty acids were administered from –day 2 to +day 2 (Table 2). There was no significant diminution of lung colonization with either pre- (–day 7 to –day 3) or post- (+day 3 to +day 7) treatment with fatty acids.

**Table 3** Preventive effect of pretreatment with unsaturated fatty acids on metastatic potential of Co 26Lu cells

| Fatty acid | Median no. of lung colonies (range) |
|---|---|
| Untreated control | 184 (150–245) |
| Linoleic acid | 114[a] (69–167) |
| EPA | 106[a] (76–156) |
| DHA | 47[b] (36–53) |

Co 26Lu tumour cells were implanted s.c. into CDF1 mice and fatty acids were administered p.o. at 0.1 ml per mouse daily from days 5 to 15. Co 26Lu cells (1 × $10^5$ cells per mouse) from five different tumours per group were injected into the tail veins of three new mice. Lung colonies were counted on day 12. [a]$P$ <0.05 compared with untreated control. [b]$P$<0.01 compared with untreated control, linoleic acid and EPA.

### Effects of fatty acids on spontaneous metastases of Co 26Lu to the lung

Orally administered EPA and DHA caused significant retardation of tumour growth of s.c. implanted Co 26Lu, especially when administered at 0.2 ml per mouse (Figure 1). However, linoleic acid did not exert any inhibitory influence, even at the high dose (data not shown). In general, daily PUFA doses of 0.2 ml per mouse were associated with loss of body weights, indicative of transient toxicity to the host. However, recover of body weight to the untreated control level was observed during treatment with PUFAs (Figure 2).

When s.c.-implanted tumour volumes were 100 $mm^3$ and over, 100% of the animals had microscopic lung metastases. The incidences of grossly visible lung metastatic nodules, which were found in all mice on day 28 in the untreated control group, were also significantly decreased in the groups administered *n*-3 PUFAs, EPA and in particular DHA (Figure 3). No inhibition of lung metastasis was found at a dose of 0.05 ml per mouse of DHA (data not shown), and administration of 0.2 ml per mouse resulted in a diminished inhibitory effect on the formation of metastatic nodules in the lung. Arachidonic acid demonstrated a tendency to decrease the number of metastatic nodules in the lung. On the other hand, linoleic acid at a daily dose of 0.2 ml per mouse promoted metastasis.

### Effects of acetylsalicylic acid and indomethacin on lung metastasis

DHA is known to inhibit cyclo-oxygenase activity (Corey et al, 1983). The effects of the cyclo-oxygenase inhibitors, acetylsalicylic acid and indomethacin, were therefore investigated. Indomethacin (1 mg $kg^{-1}$ i.p.) and acetylsalicylic acid (100 mg $kg^{-1}$ i.p.) decrease prostaglandin levels in mice (Gupta, 1989). No significant influence on the formation of metastatic nodules in the lung was observed with either of the agents alone (data not shown) or together with linoleic acid or DHA (median number of lung metastatic nodules – untreated control, 35; acetylsalicylic acid 100 mg $kg^{-1}$ i.v., 29; linoleic acid, 39; DHA, 14.5; linoleic acid + acetylsalicylic acid, 29.5; DHA + acetylsalicylic acid, 26).

### Lung colonization after intravenous injection of Co 26Lu cells obtained from mice administered fatty acids

As shown in Table 3, tumour cells obtained after exposure to DHA, EPA or linoleic acid demonstrated decreased numbers of

© Cancer Research Campaign 1997 

**Table 4** Fatty acid composition in the plasma after treatment of Co 26Lu-bearing mice with various fatty acids

| Fatty acids | Total (µg ml[-1]) | Fatty acid composition (%, mean±s.e.) | | | | | | | | |
|---|---|---|---|---|---|---|---|---|---|---|
| | | 16:0 | 18:0 | 18:1 *n*-9 | 18:1 *n*-7 | 18:2 *n*-6 | 18:3 | 20:4 *n*-6 | 20:5 *n*-3 | 22:6 *n*-3 |
| Untreated | 11 374 | 21.4 ± 0.8 | 8.1 ± 0.1 | 22.4 ± 0.5 | 3.7 ± 0.1 | 15.8 ± 0.4 | 0.4 ± 0.1 | 10.6 ± 0.1 | 0.5 ± 0.1 | 6.3 ± 0.2 |
| Oleic acid | 4195 | 21.1 ± 0.9 | 8.6 ± 0.6 | *32.2 ± 1.5*[a] | 4.2 ± 0.3 | 12.0 ± 0.5 | 0.2 ± 0.0 | 7.7 ± 0.9 | 0.3 ± 0.1 | 4.9 ± 0.5 |
| Linoleic acid | 3326 | 19.5 ± 0.8 | 9.8 ± 1.1 | 16.5 ± 1.1[a] | 2.0 ± 0.1[a] | *25.3 ± 2.2*[a] | 0.2 ± 0.0 | 14.3 ± 1.0[b] | 0.2 ± 0.0 | 4.6 ± 0.5 |
| Arachidonic acid | 4569 | 17.4 ± 0.5[a] | 7.9 ± 0.2 | 18.4 ± 1.3 | 1.3 ± 0.1[a] | 6.1 ± 0.3[a] | 0.2 ± 0.1 | *35.7 ± 1.4*[a] | 0.1 ± 0.0 | 3.8 ± 0.3 |
| EPA | 5342 | 21.0 ± 1.1 | 7.9 ± 0.2 | 20.3 ± 1.1 | 1.8 ± 0.1[a] | 10.6 ± 0.6[a] | 0.3 ± 0.2 | 3.1 ± 0.2[a] | *13.9 ± 1.7*[a] | 11.1 ± 0.8[a] |
| DHA | 5094 | 21.2 ± 0.9 | 7.0 ± 0.2 | 21.4 ± 0.7 | 1.6 ± 0.1[a] | 11.0 ± 0.5[b] | 0.6 ± 0.0 | 2.8 ± 0.3[a] | 2.9 ± 0.2 | *24.3 ± 1.7*[a] |

Tumour-bearing mice (five mice per group) were treated with unsaturated fatty acids (0.1 ml per mouse) orally once a day for ten consecutive days from day 5. Blood was taken from mice anaesthetized with ethyl ether 24 h after last treatment. The plasma was separated by centrifugation, and the samples were assayed for fatty acids by gas chromatography. [a]$P<0.01$ compared with untreated control group; [b]$P<0.05$ compared with untreated control group. Italic indicates correspondence with the administered fatty acid.

**Table 5** Fatty acid levels in Co 26Lu tumour tissue after treatment of Co 26Lu-bearing mice with various fatty acids

| Fatty acids | Total (mg g[-1]) | Fatty acid composition (%, mean±s.e.) | | | | | | | | |
|---|---|---|---|---|---|---|---|---|---|---|
| | | 16:0 | 18:0 | 18:1 *n*-9 | 18:1 *n*-7 | 18:2 n-6 | 18:3 | 20:4 n-6 | 20:5 n-3 | 22:6 n-3 |
| Untreated | 255.4 | 22.0 ± 0.4 | 6.1 ± 1.0 | 39.3 ± 1.4 | 3.9 ± 0.2 | 7.8 ± 0.4 | 0.4 ± 0.1 | 3.3 ± 0.7 | 0.1 ± 0.1 | 1.6 ± 0.5 |
| Oleic acid | 164.4 | 17.6 ± 0.8[a] | 6.9 ± 1.1 | *40.0 ± 3.7* | 4.6 ± 0.2[b] | 7.1 ± 0.2 | 0.3 ± 0.1 | 5.5 ± 1.6 | 0.1 ± 0.1 | 3.0 ± 0.8 |
| Linoleic acid | 231.6 | 21.6 ± 0.4 | 5.4 ± 0.6 | 38.8 ± 2.1 | 3.1 ± 0.2 | *12.4 ± 1.7*[a] | 0.3 ± 0.0 | 3.1 ± 0.7 | 0.0 | 1.0 ± 0.2 |
| Arachidonic acid | 198.8 | 21.3 ± 0.5 | 6.5 ± 0.9 | 39.4 ± 2.9 | 3.4 ± 0.1 | 5.9 ± 0.3 | 0.3 ± 0.0 | *6.1 ± 1.4* | 0.0 | 1.1 ± 0.3 |
| EPA | 245.5 | 23.2 ± 0.4 | 8.7 ± 1.9 | 32.3 ± 4.2 | 3.2 ± 0.1 | 5.8 ± 0.2 | 0.3 ± 0.0 | 2.2 ± 0.5 | *1.7 ± 0.1*[a] | 3.9 ± 1.0[b] |
| DHA | 137.0 | 24.1 ± 0.5 | 5.7 ± 0.3 | 40.4 ± 1.3 | 3.0 ± 0.1 | 5.5 ± 0.2 | 0.3 ± 0.0 | 1.2 ± 0.1 | 0.3 ± 0.1 | *4.3 ± 0.5*[b] |

Tumour-bearing mice (five mice per group) were treated with unsaturated fatty acids orally once a day for ten consecutive days from day 5. The tumours were taken from tumour-bearing mice 24 h after last treatment and were immediately frozen; the samples were then assayed for fatty acids by gas chromatography. [a]$P<0.01$ compared with untreated control group; [b]$P<0.05$ compared with untreated control group. Italic indicates correspondence with the administered fatty acid.

lung colonies compared with their untreated counterparts. In particular, tumour cells treated with DHA showed a marked decrease in their lung colony-forming ability ($P<0.01$) compared with those exposed to EPA or linoleic acid.

### Changes in fatty acid contents in plasma and tumour tissue following oral administration of PUFA

As shown in Table 4, oral administration of unsaturated fatty acids produced a decrease in the total fatty acids in the plasma. The arachidonic acid contents increased following administration of either arachidonic acid or linoleic acid and decreased following administration of oleic acid, EPA or DHA. Administration of EPA or DHA also caused elevations of their contents. In addition, a slight increase in EPA was observed following DHA administration, and a slight decrease in DHA was noted following linoleic acid and arachidonic acid administration.

In Co 26Lu tumour tissue (Table 5), total fatty acids did not change markedly except after DHA and oleic acid administration. Oleic acid constituted 40% of the total fatty acids. Arachidonic acid contents were low, following the administration of DHA. The EPA contents were very low, except after administration of EPA and DHA. DHA increased following administration of EPA and DHA. Accordingly, administration of unsaturated fatty acid altered the contents of fatty acids in tumour tissues. In particular, exposure to DHA caused marked changes in constituents.

### DISCUSSION

EPA and DHA did not show any inhibition of tumour growth and metastases in animals fed a normal diet rich in linoleic acid. In this study, using the AIN93M(c) diet, in which linoleic acid content was markedly reduced, DHA and EPA, especially at high doses, significantly inhibited the growth of the highly metastatic colon carcinoma Co 26Lu, as well as lowering the numbers of lung metastatic colonies. On the other hand, linoleic acid exerted an enhancing effect on metastasis at high doses.

Mammary tumour cells with a high capacity for metastasis have been reported to contain elevated levels of prostaglandins compared with their poorly metastatic counterparts (Fulton and Heppner, 1985). The eicosanoid content of tissues may clearly be influenced by the fatty acid constituents of the dietary fats consumed by the host animal. Linoleic acid and *n*-6 PUFAs are major sources of arachidonic acid, which is further metabolized to prostaglandins and leukotrienes by reactions catalysed by cyclo-oxygenase and lipoxygenase respectively. While DHA is known to inhibit cyclo-oxygenase activity (Corey et al, 1983), the failure in the present study of acetylsalicylic acid and indomethacin, which reduce prostaglandin levels (Gupta, 1989), to inhibit the formation of lung metastatic nodules, either alone or following administration of linoleic acid or DHA, suggests that the inhibition of metastasis observed for fatty acids is not dependent on their influence on production of prostaglandins. In addition, administration of arachidonic acid

 © Cancer Research Campaign 1997

itself did not result in any enhancing effect on metastasis to the lung, despite the increase in this *n*-6 fatty acid in tumour tissue. The results thus indicate that prostaglandin content does not correlate with formation of metastatic nodules in this tumour system.

The effects of PUFAs have been shown to be selective for cancer cells without affecting normal cells in vitro (Begin et al, 1986). EPA, in particular, has been reported to significantly inhibit the growth of human pancreatic cancer cell lines in vitro (Falconer et al, 1994), and this effect appears to be associated with the generation of lipid peroxides (Begin et al, 1985). In Co 26Lu culture cells in RPMI-1640 containing 10% fetal bovine serum (FBS), $IC_{50}$ values for treatment with EPA and DHA were all about 50 μM, whereas values for oleic acid and linoleic acid were over 100 μM after 6 days' exposure. There were no large differences between EPA and DHA. In our in vivo study, oral administration of EPA and DHA before or after the injection of tumour cells into a tail vein did not significantly inhibit lung colonization so that a simultaneous presence appears to be of importance. Incorporation of EPA and DHA may yield lipid peroxides, which can kill the tumour cells. However, the effect of oleic acid cannot be simply explained by the same mechanism and requires further investigation.

The ability of tumour cells to metastasize has been shown to depend on the fatty acid composition of the cell membrane and reflect the dietary fat intake (Cave and Erickson-Lucas, 1982; Cohen et al, 1986). Cells are modified by the fatty acid constituents of the culture medium in vitro (Wicha et al, 1979; Ginsberg et al, 1982), and membranes rich in linoleic acid exhibit high fluidity (Ginsberg et al, 1982; Taraboletti et al, 1989). In murine tumour cell lines, a relationship exists between metastatic ability and the lipid composition of the cell membrane, with linoleic acid playing an important role (Kier and Franklin, 1991). Different contents of oleic acid and arachidonic acid have been noted between primary and metastatic tumours (Kier et al, 1988). In metastatic variants of a series of human prostatic adenocarcinoma cell lines, the arachidonic acid level was found to be significantly decreased (Dahiya et al, 1992). However, in our study, tumour cells obtained from the hosts administered EPA or DHA demonstrated decreases in both arachidonic acid contents and metastatic ability. Moreover, i.v. injection of tumour cells with a greatly increased content of linoleic acid did not result in any increase in lung metastatic nodules. Administration of large amounts of fatty acids (0.2 ml) caused toxicity, and this was associated with some reduction in the inhibitory effects on lung metastatic nodules, presumably owing to an impaired host defence system, such as inhibition of macrophage activation by DHA (Dustin et al, 1990). In conclusion, the present data suggest that fatty acid composition of tumour cell membrane is an important factor with relevance to metastatic potential. An increase in *n*-3 PUFAs, such as EPA and DHA, appears to reduce these features of malignancy without necessarily blocking prostaglandin synthesis. The present model should allow light to be cast on the mechanisms underlying this beneficial influence.

### ACKOWLEDGEMENT

This study was supported in part by a grant from the Grant-in-Aid for Cancer Research from the Ministry of Health and Welfare.

### REFERENCES

Awad AB and Spector AA (1976) Modification of the fatty acid composition of Ehrlich ascites tumor cell plasma membranes. *Biochim Biophys Acta* **426**: 723–731

Begin ME, Das UN, Ells G and Horrobin DF (1985) Selective killing of human cancer cells by polyunsaturated fatty acids. *Prostaglandins Leukotrienes Med* **19**: 177–186

Begin ME, Ells G, Das UN and Horrobin DF (1986) Differential killing of human carcinoma cells supplemented with n-3 and n-6 polyunsaturated fatty acids. *J Natl Cancer Inst* **77**: 1053–1062

Bertomeu MC, Gallo S, Lauri D, Haas TA, Orr FW, Bastida E and Buchanan MR (1993) Interleukin 1-induced cancer cell/endothelial cell adhesion in vitro and its relationship to metastasis in vivo: role of vessel wall 13-HODE synthesis and integrin expression. *Clin Exp Metastasis* **11**: 243–250

Bligh EG and Dyer WJ (1959) A rapid method of total lipid extraction and purification. *Can J Biochem Physiol* **37**: 911–917

Cave WT, Jr and Erickson-Lucas MJ (1982) Effects of dietary lipids on lactogenic hormone receptor binding in rat mammary tumors. *J Natl Cancer Inst* **68**: 319–324

Chen YQ, Liu B, Tang DG and Honn KV (1992) Fatty acid modulation of tumor cell-platelet-vessel wall interaction. *Cancer Metastasis Rev* **11**: 389–409

Cohen LA, Thompson DO, Choi K, Karmali RA and Rose DP (1986) Dietary fat and mammary cancer. II. Modulation of serum and tumor lipid composition and tumor prostaglandins by different dietary fats: association with tumor incidence patterns. *J Natl Cancer Inst* **77**: 43–51

Corey EJ, Shih C and Cashman JR (1983) Docosahexaenoic acid is a strong inhibitor of prostaglandin but not leukotriene biosynthesis. *Proc Natl Acad Sci USA* **80**: 3581–3584

Dahiya R, Boyle B, Goldberg BC, Yoon W-H, Konety B, Chen K, Yen T-S B, Blumenfeld W and Narayan P (1992) Metastasis-associated alterations in phospholipids and fatty acids of human prostatic adenocarcinoma cell lines. *Biochem Cell Biol*, **70**: 548–554

Dustin LB, Shea CM, Soberman RJ and Lu CY (1990) Docosahexaenoic acid, a constituent of rodent fetal serum and fish oil diets, inhibits acquisition of macrophage tumoricidal function. *J Immunol*, **144**: 4888–4897

Falconer JS, Ross JA, Fearon KCH, Hawkins RA, O'Riordain MG and Carter DC (1994) Effect of eicosapentaenoic acid and other fatty acids on the growth *in vitro* of human pancreatic cancer cell lines. *Br J Cancer* **69**: 826–832

Fulton AM and Heppner GH (1985) Relationships of prostaglandin E and natural killer sensitivity to metastatic potential in murine mammary adenocarcinomas. *Cancer Res* **45**: 4779–4784

Ginsberg BH, Jabour J and Spector AA (1982) Effect of alterations in membrane lipid unsaturation on the properties of the insulin receptor of Ehrlich ascites cells. *Biochim Biophys Acta* **690**: 157–164

Gupta C (1989) The role of prostaglandines in masculine differentiation: modulation of prostaglandin levels in the differentiating genital tract of the fetal mouse. *Endocrinology* **124**: 129–133

Iigo M, Tsuda H and Moriyama M (1994) Enhanced therapeutic effects of anti-tumour agents against growth and metastasis of colon carcinoma 26 when given in combination with interferon and interleukin-2. *Clin Exp Metastasis* **12**: 368–374

Kier AB and Franklin C (1991) Membranes of high- and low-metastatic L tumor cell variants. *Invasion Metastasis* **11**: 25–37

Kier AB, Parker MT and Schroeder F (1988) Local and metastatic tumor growth and membrane properties of LM fibroblasts in athymic (nude) mice. *Biochem Biophys Acta* **938**: 434–446

Kort WJ, Weijma IM, Bijma AM, van Schalkwijk WP, Vergroesen AJ and Westbroek DL (1987) Omega-3 fatty acids inhibiting the growth of a transplantable rat mammary adenocarcinoma. *J Natl Cancer Inst* **79**: 593–599

Rao GA and Abraham S (1976) Enhanced growth rate of transplanted mammary adenocarcinoma induced in C3H mice by dietary linoleate. *J Natl Cancer Inst* **56**: 431–432

Raz A and Ben-Ze ev A (1987) Cell-contact and -architecture of malignant cells and their relationship to metastasis. *Cancer Metastasis Rev* **6**: 3–21

Rose DP and Connolly JM (1993) Effects of dietary omega-3 fatty acids on human breast cancer growth and metastases in nude mice. *J Natl Cancer Inst* **85**: 1743–1747

Rose DP and Hatala MA (1994) Dietary fatty acids and breast cancer invasion and metastasis. *Nutr Cancer* **21**: 103–111

Rose DP, Hatala MA, Connolly J M and Rayburn J (1993) Effect of diets containing different levels of linoleic acid on human breast cancer growth and lung metastasis in nude mice. *Cancer Res* **53**: 4686–4690

Schroeder F (1984) Fluorescence probes in metastatic B16 melanoma membranes. *Biochim Biophys Acta* **776**: 299–312

Taraboletti G, Perin L, Bottazzi B, Mantovani A, Giavazzi R and Salmona M (1989) Membrane fluidity affects tumor-cell motility, invasion and lung-colonizing potential. *Int J Cancer* **44**: 707–713

Wicha MS, Liotta LA and Kidwell WR (1979) Effects of free fatty acids on the growth of normal and neoplastic rat mammary epithelial cells. *Cancer Res* **39**: 426–435

© Cancer Research Campaign 1997